# Comparison of Risk of Stroke in Patients With and Without Depression: A Systematic Review and Meta-Analysis

**DOI:** 10.7759/cureus.53057

**Published:** 2024-01-27

**Authors:** Manisha Kanumuri, Areeba Khan, Asfia Neshat, Goutham Alapati, Gopi Sairam Reddy Mulaka, Nimra Nisar, Saima Batool, FNU Arti

**Affiliations:** 1 Psychiatry, MediCiti Institute of Medical Sciences, Hyderabad, IND; 2 Critical Care Medicine, United Medical and Dental College, Karachi, PAK; 3 Internal Medicine, Connolly Hospital, Dublin, IRL; 4 Oncology, St. Martinus University Faculty of Medicine, Willemstad, CUW; 5 Internal Medicine, St. Martinus University Faculty of Medicine, Willemstad, CUW; 6 Internal Medicine, Allama Iqbal Medical College, Lahore, PAK; 7 Internal Medicine, Hameed Latif Hospital, Lahore, PAK; 8 Medicine, Muhammad Mahar Medical College, Sukkur, PAK

**Keywords:** systematic review and meta analysis, major depressive disorder, risk, stroke, depression

## Abstract

Individuals with depression face an elevated stroke risk, marked by an unfavorable prognosis. This meta-analysis aims to determine the impact of depression on stroke risk. The current meta-analysis was conducted using the guidelines established by the Preferred Reporting Items for Systematic Reviews and Meta-Analyses (PRISMA). We selected studies through a systematic review of electronic databases, including PubMed, EMBASE, and CINAHL from January 2011 to January 2023. Google Scholar was utilized to identify supplementary studies. Furthermore, we scrutinized citation lists of reported articles for additional potential studies. Only English-language articles were included in the review. A total of 15 studies were included in this meta-analysis. The pooled sample size was 744,179. Sample size of the included studies ranged from 560 to 487,377. The pooled estimate of 15 studies showed that the risk of stroke was 1.47 times higher in individuals with depression compared to the individuals without depression, and the difference is statistically significant (RR: 1.47, 95% CI: 1.30 to 1.66, p-value<0.001). Age and hypertension emerged as significant predictors of stroke risk in depressed individuals identified through meta-regression. These findings underscore the importance of targeted preventive strategies for depression-related stroke risk, especially considering age-specific considerations and associated factors.

## Introduction and background

Major depression, a mental disorder leading to disability, stands among the most incapacitating conditions in the European Union [1]. An estimated 3.8% of the population experience depression, including 5% of adults and 5.7% of adults older than 60 years. Approximately 280 million people in the world have depression [2]. The economic burden of depressed patients rose by around 20% from 2005 to 2010 [3]. Notably, the growing cost of depression management is predominantly attributed to expenses associated with depression-related comorbidities [4].

Individuals with depression face an elevated stroke risk, marked by an unfavorable prognosis [5]. Stroke, a leading cause of substantial long-term disability and mortality, saw a slower decline in age-standardized incidence, mortality, and disability-adjusted life-year rates globally from 2010 to 2019 compared to the previous decade [6]. Those with depressive symptoms demonstrated an increased risk of both ischemic and hemorrhagic stroke, along with poorer post-stroke recovery [7]. Key risk factors for depression in stroke patients and the general population include older age, female gender, single cohabitation status, limited educational attainment, diabetes, high somatic comorbidity, history of depression, and stroke severity [8].

A meta-analysis conducted by Dong et al. [9] reported that depression is associated with increased risk of stroke. In contrast, several studies failed to establish depression as a significant risk factor for cerebrovascular diseases [10-11]. The relationship between depression and an increased risk of stroke is complex and multifaceted. Depression is associated with increased inflammation in the body. Chronic inflammation is known to contribute to the development of atherosclerosis (hardening and narrowing of arteries), which is a major risk factor for stroke [12].

Building upon the observation that various research, including prospective studies, has explored the connections between depressive disorder, stroke occurrence, and mortality, generating divergent outcomes, it is essential to delve into the implications and potential reasons behind these divergences. This divergence may stem from several factors, such as differences in study design, sample characteristics, measurement tools, and statistical methodologies. To address these divergent outcomes meaningfully, we conducted a meta-analysis or systematic review, which involves synthesizing data from multiple studies to identify patterns and trends. The aim of this meta-analysis is to determine the impact of depression on the risk of stroke.

## Review

Methodology

The current systematic review or meta-analysis was conducted using the guidelines established by the Preferred Reporting Items for Systematic Reviews and Meta-Analyses (PRISMA).

We selected studies through a systematic review of electronic databases, including PubMed, EMBASE, and CINAHL from January 2011 to January 2023. Google Scholar was utilized to identify supplementary studies. Furthermore, we scrutinized citation lists of reported articles for additional potential studies. The search employed keywords such as "depression" and "stroke," along with Medical Subject Heading (MeSH) terms, synonyms, and Boolean operators. the PubMed search strategy is as follows: "(Depression[Mesh] OR Major Depressive Disorder[Mesh] OR depressive disorder [Mesh] OR MDD[Mesh] OR depressive disorder [Mesh]) AND (Stroke[Mesh] OR Cerebrovascular Accident[Mesh] OR Cerebral Infarction[Mesh] OR Brain Ischemia[Mesh])."

Our focus was on identifying studies investigating the link between depression and incident stroke. Only English-language articles were included in the review. Two evaluators independently examined the titles and abstracts of each study. The full texts of the studies were then accessed to determine if they met the eligibility criteria before proceeding with data extraction. Any discrepancies between the two reviewers were resolved through consensus or discussion, involving a third investigator if necessary.

Eligibility Criteria

Based on our structured PECOS (participants, exposure, comparators, outcomes, and study design) query, we included an original epidemiological study if it met the following criteria: (1) involved adults aged 18 years or older, (2) defined the exposure variable as depression, with depression assessment conducted prospectively at baseline using an objectively measured validated scale or diagnostic criteria, (3) had a comparison group comprising participants without depression at the study’s outset, and (4) focused on the dichotomous outcome event of interest, which was the occurrence of a first-ever stroke during the follow-up period, encompassing fatal and nonfatal ischemic stroke, transient ischemic attack (TIA), and intracerebral hemorrhage (referred to as "all stroke"). Meta-analyses, reviews, and editorials were excluded from consideration.

Quality Assessment and Data Processing

Data retrieval utilized predetermined forms, encompassing details such as the first author, publication year, country, sample size, follow-up duration, and instrument used to assess depression. Two independent investigators performed data extraction from each eligible article, resolving discrepancies through consensus with the principal author. Quality assessment of the included studies was performed using the Newcastle-Ottawa Scale (NCOS). It is a widely used tool for assessing the quality of non-randomized studies.

The statistical evaluation utilized the Cochrane Collaboration Review Manager Software (RevMan version 5.4.1) and STATA version 16.0 (Version 16, StataCorp, College Station, TX). The analysis employed the random-effects meta-analysis model, and presentation of the treatment effect was achieved through forest plots, depicting the risk ratio (RR) alongside its corresponding 95% confidence interval (CI). The random-effects model was employed to address heterogeneity among the study results, stemming from variations in study design, population characteristics, and the tools utilized for assessing depression, among other factors. Statistical significance was defined as a p-value of ≤0.05. To measure inconsistency, I^2^ statistics were utilized, and Cochran's Q test was employed for statistically testing heterogeneity. Heterogeneity was considered significant if the p-value was below 0.1. Meta-regression was performed to systematically explore and examine potential sources of heterogeneity observed across studies in a systematic review or meta-analysis. For the assessment of publication bias, Egger’s regression test was applied, with a p-value of ≤0.05 considered significant for detecting publication bias.

Results

**Figure 1:**
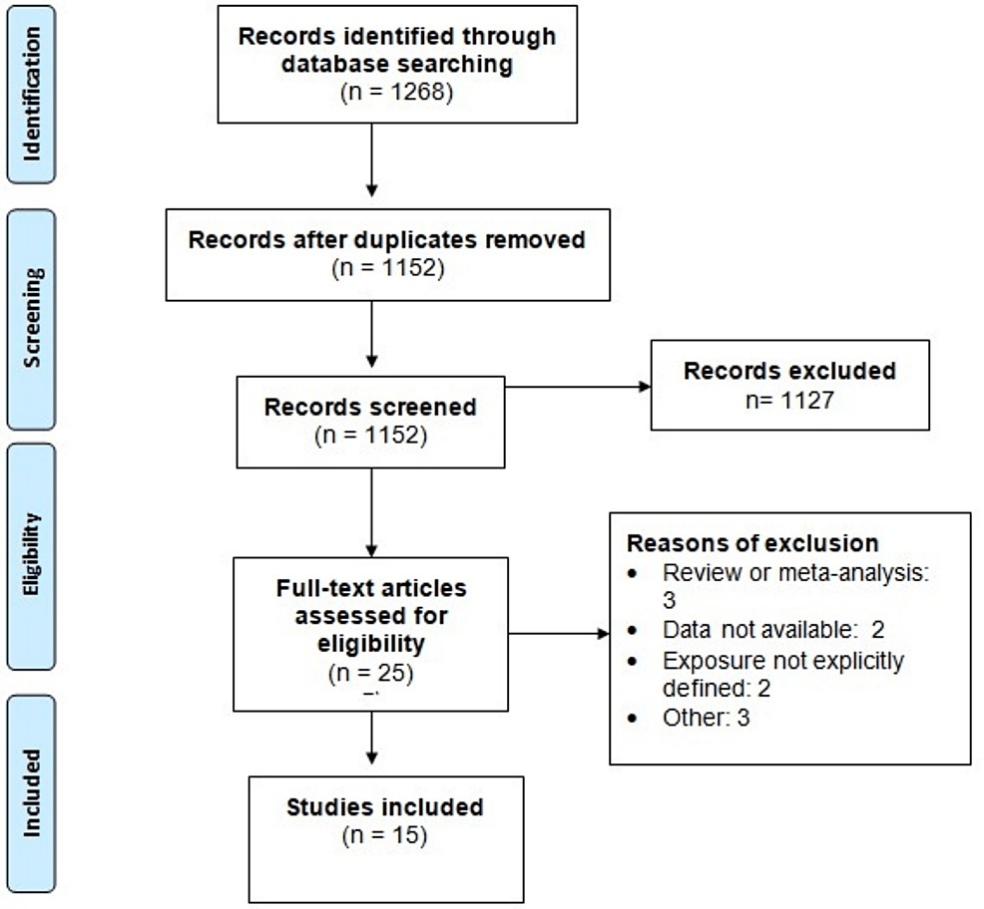
PRISMA flowchart of study selection PRISMA, Preferred Reporting Items for Systematic Reviews and Meta-Analyses

Through online database searching, we obtained 1,268 articles (PubMed: 552, EMBASE: 386, CINAHIL: 330). After removing duplicates, 1,152 studies were initially screened using titles and abstracts. After initial screening, 1,127 studies were thoroughly screened using full-text review. Finally, 15 studies were included in this meta-analysis. Figure 1 shows the process of study selection.

Study Characteristics

Table 1 shows the characteristics of the included studies. The pooled sample size was 74,4179. Sample size of the included studies ranged from 560 to 487,377. Depression or depressive symptoms were ascertained by screening scales in most included studies including the Center for Epidemiologic Studies Depression Scale (CES-D), the depression subscale of the 30-item General Health Questionnaire (GHQ), International Classification of Diseases (ICD)-9, ICD-7, Mental Health Inventory-5 (MHI-5), and Multinational Monitoring of Trends and Determinants of Cardiovascular Disease (MONICA)-Psychology (MOPSY). Stroke was ascertained from medical records or death certificates in most studies, with some combining these with self-reported measures. Table 2 presents risk-of-bias assessment of the included studies. Table 2 presents the quality assessment of the included studies.

**Table 1:** Characteristics of the included studies GHQ, General Health Questionnaire; Center for Epidemiologic Studies Depression Scale; MOPSY, Multinational Monitoring of Trends and Determinants of Cardiovascular Disease (MONICA)-Psychology; ICD, International Classification of Diseases; MHI-5, Mental Health Inventory-5; CIDI, Composite International Diagnostic Interview

Study	Year	Sample size	Country	Follow-up (years)	Instrument
Brunner et al. [13]	2014	31,395	United Kingdom	5	GHQ-30
Cui et al. [14]	2021	10,100	China	5	CES-D
Everson-Rose et al. [15]	2014	2,658	United States	8.5	CES-D
Gafarov et al. [16]	2013	560	Russia	16	MOPSY
Gilsanz et al. [17]	2017	4,319	United States	9	CES-D
Jackson and Mishra [18]	2013	10,399	Australia	12	CES-D
Li et al. [19]	2012	5,015	Taiwan	9	ICD-9
Majed et al. [20]	2012	8,746	France	10	CES-D
Murphy et al. [21]	2023	26,877	Multinational	NR	CIDI
Pan et al. [22]	2011	80,574	United States	6	MHI-5
Pequignot et al. [23]	2013	7,308	France	5.3	CES-D
Rahman et al. [24]	2013	31,699	Sweden	3.9	ICD-7
Sico et al. [25]	2021	33,628	United States	9.2	ICD-9
Sun et al. [26]	2016	487,377	China	7.2	Composite International Diagnostic Inventory-Short Form
Zahodne et al. [27]	2017	3,524	United States	6.4	CES-D

**Table 2:** Quality assessment of the included studies

Study	Selection	Comparability	Exposure/outcome assessment	Overall
Brunner et al. [13]	3	2	3	Good
Cui et al. [14]	3	2	3	Good
Everson-Rose et al. [15]	3	2	4	Good
Gafarov et al. [16]	2	2	3	Good
Gilsanz et al. [17]	3	1	3	Fair
Jackson and Mishra [18]	3	2	3	Good
Li et al. [19]	3	2	3	Good
Majed et al. [20]	2	2	3	Good
Murphy et al. [21]	2	1	3	Fair
Pan et al. [22]	3	2	3	Good
Pequignot et al. [23]	3	2	4	Good
Rahman et al. [24]	3	1	3	Fair
Sico et al. [25]	3	2	4	Good
Sun et al. [26]	2	2	3	Fair
Zahodne et al. [27]	3	2	3	Good

Comparison of Risk of Stroke Between Individuals With Depression and Without Depression

**Figure 2:**
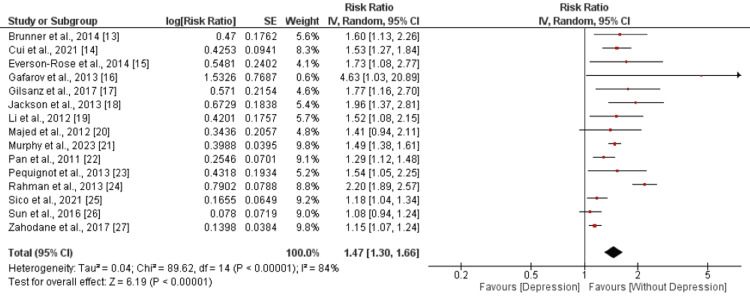
Comparison of risk of stroke Sources: [12-27]

All 15 studies compared the risk of stroke. The pooled estimate of 15 studies showed that the risk of stroke was 1.47 times higher in individuals with depression compared to the individuals without depression, and the difference was statistically significant (RR: 1.47, 95% CI: 1.30 to 1.66, p-value<0.001), as shown in Figure 2. Significant heterogeneity was reported among the study results (I^2^: 84%). All 15 included studies reported a positive association between depression and stroke. Out of 15 studies, two did not report significant association (p-value>0.05).

Meta-Regression

The meta-regression analysis examined the relationship between several covariates (age, gender, diabetes, hypertension, body mass index [BMI], smoking) and the risk of stroke. However, gender, diabetes, and BMI did not demonstrate statistically significant associations, indicating that gender, diabetes, and BMI are not identified as significant predictors of the risk of stroke based on the analyzed studies. However, age and hypertension were identified as the predictors of stroke (Table 3).

**Table 3:** Results of meta-regression BMI, body mass index; CI, confidence interval

Covariate	Number of studies	Coefficient	95% CI	P-value
Age	12	0.06	0.01 to 0.15	0.025
Gender (male)	12	0.0002	-0.0008 to 0.0004	0.569
Diabetes	9	0.0004	-0.0002 to 0.0007	0.386
Hypertension	9	0.02	0.001 to 0.06	0.002
BMI	8	0.0005	-0.0003 to 0.002	0.712
Current smoker	5	0.01	-0.05 to 0.26	0.002

Subgroup Analysis

We performed subgroup analysis on the basis of follow-up duration, sample size, number of males, and number of publications to assess depression; the results are shown in Table 4. For studies with shorter follow-up durations (≤6 years), the RR was 1.61 (95% CI: 1.27 to 2.04), whereas in studies with longer follow-up (>6 years), the RR was slightly lower at 1.32 (95% CI: 1.16 to 1.51) and the difference between these two groups was insignificant. Interestingly, the risk of stroke appeared to differ based on publication year, with a higher RR of 1.67 (95% CI: 1.37 to 2.03) for studies published before 2015 compared to a lower RR of 1.30 (95% CI: 1.13 to 1.49) for those published after 2015. Additionally, the subgroup analysis stratified by sample size suggested consistent RRs (1.47) for both smaller (<10000) and larger (≥10,000) sample sizes. Notably, gender-based stratification indicated higher RRs among studies with a lower percentage of males (≤30% males: RR = 1.86, 95% CI: 1.26 to 2.75), whereas studies with higher proportions of males (>60% males: RR = 1.38, 95% CI: 1.17 to 1.63) exhibited a comparatively lower but still significant risk of stroke associated with depression.

**Table 4:** Findings of subgroup analysis RR, risk ratio; CI, confidence interval

Groups	Categories	RR (95% CI)	I^2^	P-value
Follow-up	≤6 years	1.61 (1.27 to 2.04)	85%	0.15
	>6 years	1.32 (1.16 to 1.51)	63%	
Publication year	≤2015	1.67 (1.37 to 2.03)	73%	0.04
	>2015	1.30 (1.13 to 1.49)	86%	
Sample size	<10,000	1.47 (1.20 to 1.80)	57%	0.97
	≥10,000	1.47 (1.26 to 1.66)	89%	
Gender	≤30% males	1.86 (1.26 to 2.75)	89%	0.39
	30-60% males	1.44 (1.13 to 1.83)	63%	
	>60% males	1.38 (1.17 to 1.63)	70%	

Discussion

The primary outcome from this meta-analysis involving nearly 744,179 participants, in line with prior analyses, indicates a 47% elevated risk of experiencing a first-ever stroke among individuals with depression. Additionally, our findings align with recently published pooled estimates, affirming an overall greater risk of stroke [28], although our study incorporates new studies.

The relationship between depression and the risk of stroke is complex and involves various factors. Elevated inflammation, disturbances in the immune system, disruptions in the hypothalamic-pituitary-adrenal axis, increased activation of platelets, and alterations in coagulation are all factors that play a role [29]. Lifestyle elements, including an unhealthy diet and lack of physical activity, may further contribute to the risk. Moreover, depression can influence medication adherence, affecting the management of other risk factors related to stroke [30]. Understanding these interconnected mechanisms is vital for a thorough approach to stroke prevention in individuals dealing with depression.

On the contrary, Barlinn et al. [28] conducted a meta-analysis aiming to determine whether individuals with depression, without cardiovascular or cerebrovascular issues, have a higher likelihood of experiencing a stroke, or if this association could be attributed to reverse causality. Among the 28 studies they examined (involving 681,139 patients), only seven studies utilized the Diagnostic and Statistical Manual of Mental Disorders (DSM) or ICD criteria for diagnosing depression. Their findings suggested that the elevated stroke risk associated with depression does not appear to be driven by underlying cardiovascular or cerebrovascular disorders, whether silent or clinically apparent. However, it is noteworthy that a majority of studies in this meta-analysis relied on self-reported questionnaires, introducing the possibility of misclassification and measurement bias [28]. Additionally, the inclusion of most studies in our meta-analysis that appropriately adjusted for established vascular risk factors further supports the idea that the link between depression and stroke might be independent of concurrent vascular diseases.

In our meta-regression analysis, we observed a positive correlation between advancing age and an augmented risk of stroke in individuals with depression. This finding aligns with the original vascular depression concept, proposing a specific link between depression in the elderly and cerebral small vessel disease. A recent meta-analysis further substantiated this connection, revealing a fourfold higher prevalence of cerebral small vessel disease in individuals with late onset depression compared to those with early onset depression [31]. Notably, the development of cerebral small vessel disease intensifies with age and is associated with an increased susceptibility to stroke [32]. Therefore, considering the vascular depression concept, it is reasonable to anticipate an elevated risk of stroke, particularly among elderly individuals experiencing depression. Data from the Framingham Heart Study supported this notion, demonstrating a significant fourfold increase in the risk of a first-ever ischemic stroke in individuals under 65 years with depressive symptoms, but no such association was shown in those aged 65 years or older. The observed variation in stroke risk, where it remains balanced or even increases in younger depressed individuals while rising with age in the general population, may be partly attributed to lower medication adherence among individuals with depression, potentially compromising primary stroke prevention [33-34].

The occurrence rate of major depressive disorder among adults aged 65 years and above varies between 1% and 5% in both U.S. and international studies [35]. Additionally, around 15% of older adults residing in the community exhibit significant depressive symptoms [36]. However, individuals in this age group experiencing depression might attribute their symptoms to medical issues or the natural aging process, potentially hindering effective treatment [37]. This challenge is further compounded by potential stigma and anxiety related to the adverse effects of antidepressants [38]. Together, these factors may lead to underreporting and the overlooking of treatment for depressive symptoms in older individuals.

Study limitations

Our quantitative synthesis of data has inherent limitations primarily tied to the clinical and methodological diversity across the included studies, as evident in the moderate heterogeneity within our pooled dataset. The clinical heterogeneity arises from the diverse self-reporting mood scales employed in the studies, potentially introducing misclassification and information bias. Another drawback is that most published studies defined outcome measures as any stroke subtype rather than focusing on specific subtypes (e.g., ischemic or hemorrhagic stroke) or etiology. Consequently, our synthesized analysis may fail to detect stroke subtype- or etiology-specific differences in the association between depression and stroke. This limitation is noteworthy, especially considering that in young adults, stroke causes are less commonly attributed to cerebrovascular diseases than in the elderly, encompassing a range of nonatherosclerotic causes. Examples include arterial dissection, hypercoagulable state, nonatherosclerotic arteriopathies, or cryptogenic causes. Therefore, our observation of a balanced stroke risk across different age groups should be interpreted cautiously. This caution extends to hemorrhagic stroke, given its varied age-dependent etiologies.

## Conclusions

In conclusion, our meta-analysis involving 15 studies and nearly 744,179 participants establishes a robust association between depression and a 47% increased risk of first-ever stroke. Despite study heterogeneity, age and hypertension emerged as significant predictors of stroke risk in depressed individuals identified through meta-regression. These findings underscore the importance of targeted preventive strategies for depression-related stroke risk, especially considering age-specific considerations and associated factors. Additional rigorous investigations are required to comprehensively address confounding variables and elucidate the underlying causal pathways connecting depressive disorders or symptoms to stroke.
